# Epilepsy

**Published:** 1855-04

**Authors:** 


Epilepsy.—Dr. Sieveking read a paper to the Harvean Society, on the Pathology and Treatment of Epilepsy; and commenced by alluding to the centric and ex-centric nature of the complaint. He did not, however, look upon it as a disease, but as a symptom. Several illustrative cases were then mentioned, and especially one occurring after a mild attack of scarlatina, in which the fits were very severe, and were stopped by the employment of anodynes. A table of cases was next brought forward, to show the predominance of the left side during the fit. Dr. Sieveking then spoke of the use of the cotyledon umbilicus in the treatment of this affection, and brought forward several cases in proof of its beneficial effects. It was described as producing a slightly sedative and diuretic action, though its exact *modus operandi* has not yet been discovered; it, however, appeared to him to exercise some control over the disease, and at the same time, was inocuous to the constitution of the patient.—*London Lancet.*

